# Graphene Inks Printed by Aerosol Jet for Sensing Applications: The Role of Dispersant on the Inks’ Formulation and Performance

**DOI:** 10.3390/s23167151

**Published:** 2023-08-13

**Authors:** Ahmad Al Shboul, Mohsen Ketabi, Daniella Skaf, Audithya Nyayachavadi, Thierry Lai Fak Yu, Tom Rautureau, Simon Rondeau-Gagné, Ricardo Izquierdo

**Affiliations:** 1 Department of Electrical Engineering, École de Technologie Supérieure, Montréal, QC H3C 1K3, Canadatrautureau25@gmail.com (T.R.); 2 Department of Chemistry and Biochemistry, Advanced Materials Centre of Research, University of Windsor, Windsor, ON N9B 3P4, Canadanyayacha@uwindsor.ca (A.N.); simon.rondeau-gagne@uwindsor.ca (S.R.-G.)

**Keywords:** aerosol jet printing, gelatin, graphene, ink, triton X-100, tween-20

## Abstract

This study presents graphene inks produced through the liquid-phase exfoliation of graphene flakes in water using optimized concentrations of dispersants (gelatin, triton X-100, and tween-20). The study explores and compares the effectiveness of the three different dispersants in creating stable and conductive inks. These inks can be printed onto polyethylene terephthalate (PET) substrates using an aerosol jet printer. The investigation aims to identify the most suitable dispersant to formulate a high-quality graphene ink for potential applications in printed electronics, particularly in developing chemiresistive sensors for IoT applications. Our findings indicate that triton X-100 is the most effective dispersant for formulating graphene ink (GTr), which demonstrated electrical conductivity (4.5 S·cm^−1^), a high nanofiller concentration of graphene flakes (12.2%) with a size smaller than 200 nm (<200 nm), a low dispersant-to-graphene ratio (5%), good quality as measured by Raman spectroscopy (I_D_/I_G_ ≈ 0.27), and good wettability (θ ≈ 42°) over PET. The GTr’s ecological benefits, combined with its excellent printability and good conductivity, make it an ideal candidate for manufacturing chemiresistive sensors that can be used for Internet of Things (IoT) healthcare and environmental applications.

## 1. Introduction

The growing interest in printed and flexible electronics for Internet of Things (IoT) applications has led to a surge in research on high-performance conductive inks [1]. As the cornerstone of printed electronics, the global market for conductive inks is projected to reach USD 3.98 billion by 2028, with a compound annual growth rate (CAGR) of 4% between 2021 and 2028 [2]. Graphene-based inks, owing to graphene’s exceptional qualities [3], have emerged as one of the most researched materials in this field [4,5]. Graphene also offers significant engineering potential, such as chemical and physical functionalization, metal coating, nanoparticle decorating, and other techniques that can tailor its properties to various devices [5].

Liquid-phase exfoliation (LPE) is widely used for preparing graphene inks [3,6]. This low-cost approach directly exfoliates high-quality graphene from raw graphite into a solvent phase [6]. While LPE can be performed in pure solvents for dispersant-free dispersions, dispersants are often required to achieve surface energy equilibrium between the graphene and the solvent [3]. This stabilizes the graphene flakes in dispersions, as graphene has a surface energy of 46.7 mJ·m^−2^ and Hansen solubility parameters (HSPs) of 18, 9.3, and 7.7 MPa^1/2^ for the dispersive (δ_D_), polar (δ_P_), and H-bond (δ_H_) parameters, respectively [7].

Dispersant-free dispersions of graphene rapidly form in solvents such as dimethylformamide (DMF) [8], cyclohexanone (Cy) [9], and N-methyl-2-pyrrolidone (NMP) [10], owing to the close match between their surface tension (30–40 mJ·m^−2^) and HSPs (17–18 MPa^1/2^ for δ_D_, 5–12 MPa^1/2^ for δ_P_, and 5–26 MPa^1/2^ for δ_H_) and those of graphene [11]. However, despite being good candidates for preparing colloidally stable graphene inks, challenges related to printability, low adhesion, high polarity, viscosity, slow drying rate, and high energy consumption make them challenging to deploy in practical applications [7]. Furthermore, their toxicity is a serious concern that must be addressed before widespread deployment in industries where sanitary conditions are crucial [3,6,12].

The growing concerns about environmental and health issues have diverted researchers’ attention to using green solvents and dispersants in the inks’ formulation [3,6,13,14,15,16,17,18]. Using such materials reduces workers’ exposure to hazardous chemicals, which can harm their health, and produces recyclable and/or biodegradable end-products, reducing the accumulation of e-waste in nature. Moreover, they can restrict the release of toxic and dangerous compounds into the environment during printed films’ degradation.

Researchers have recently focused on synthesizing graphene inks in various user-friendly solutions, such as Coca-Cola [6], an instant Nescafe solution [15], whiskey [13], a solvent mixture of ethanol and water [3], and natural oil-in-water emulsification agents [16]. These solutions offer a promising alternative to hazardous solvents while providing the necessary properties to disperse and stabilize graphene flakes in ink. In addition, scientists have explored biocompatible and biodegradable materials for graphene formulations, such as polyurethane [19,20], polylactide-co-glycolide [21], gelatin methacrylamide [22], gelatin methacrylate [23], and poly(ε-caprolactone) [24,25]. These materials effectively provided suitable rheological properties and printing quality while offering biocompatibility and biodegradability. These recent developments in synthesizing graphene inks demonstrate a shift towards more sustainable and environmentally friendly ink formulations.

**Figure 1:**
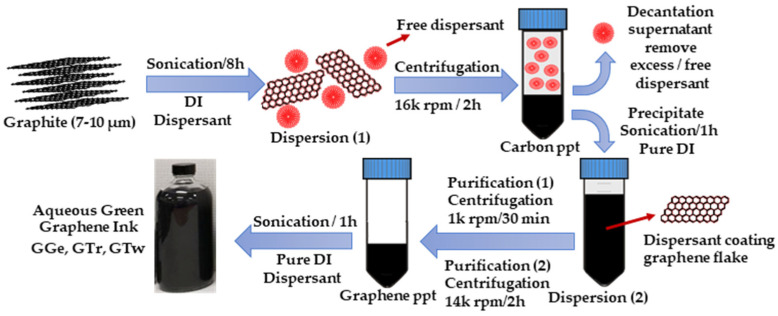
A schematic representation of the preparation process of graphene inks.

This study aims to develop aqueous graphene inks, namely GGe, GTr, and GTw. The inks were prepared using the LPE technique (Figure 1), and their binding to eco-dispersants (gelatin, triton X-100, and tween-20) was evaluated. The dispersants’ chemical structure is shown in Figure S1. Gelatin is a renewable biomaterial with excellent biocompatibility and tunable properties, making it an attractive choice for biomedical applications [26,27]. It is also a proton-conducting polymer electrolyte, making it suitable for producing biodegradable energy units [28,29]. Alternatively, triton X-100 and tween-20 are eco-surfactants that in low quantities have been used in various graphene inks to lower surface tension [30,31], resulting in improved printability. Furthermore, they effectively exfoliate graphene, resulting in dispersions with a concentration of ~20 µg·mL^−1^ [32]. These graphene inks have already been utilized in various applications, such as fabricating sensors [33,34], and energy units [30].

The dispersants used in this study are amphiphilic molecules with both hydrophilic and hydrophobic properties, and they adsorb similarly on the graphene surface. However, the dispersants’ average molecular weight (g·mol^−1^), critical micelle concentration (CMC), and other properties differ (Table S1), which can lead to variations in the properties of the resulting graphene inks. Thus, we researched the dispersants’ effects on the qualities of graphene dispersions and inks. We investigated various aspects of dispersion, including the optimal dispersant concentration for the highest graphene concentration, the distribution of graphene flake size and thickness, and the net dispersant-to-graphene ratio. Using an aerosol jet printing (AJP) process, we also examined the effect of dispersants on the inks’ printability, adhesion over polyethylene terephthalate (PET) substrate, film smoothness, net electrical conductivity, electrochemical characteristics, and mechanical properties.

The decision to use AJP was based on a few key benefits of this printing technique. First, AJP is a popular, noncontact, digital additive manufacturing process that offers high-resolution printing capabilities and works with a wide range of functional materials [35]. AJP achieves high-resolution printing with fine patterns, with a printing resolution of approximately 10 μm [36]. Its versatility extends to fine-feature patterning with feature sizes from 10 nm to 10 mm on rigid and flexible substrates [37], giving us greater flexibility in precise thin film formation. Finally, AJP offers large nozzle diameters (e.g., 150 µm and 300 µm), effectively preventing potential clogging issues caused by materials like graphene flakes with sizes in the µm range [38]. These key points made AJP an appealing choice for printing graphene inks and fabricating sensors in general, as demonstrated in the most recent reports (Table S2) [35,38,39,40,41,42,43,44,45,46]. Its notable advantages include high precision in graphene deposition, making it suitable for a wide range of sensor applications. The technique’s versatility and demonstrated success have further elevated its attractiveness for advanced sensor fabrication.

## 2. Materials and Methods

### 2.1. Materials and Reagents

Graphite (7–10 µm) was sourced from Alfa Aesar (Ward Hill, MA, USA), gelatin was supplied by Sigma-Aldrich (Waltham, MA, USA), triton X-100 was obtained from Millipore Sigma (Oakville, ON, Canada), and tween-20 from BioShop Canada. We prepared all the solutions with deionized water (DI) with a resistivity of >18 MΩ·cm^−1^.

### 2.2. Optimizing Graphene Dispersions

Aqueous graphene dispersions were created using the LPE technique. First, we prepared aqueous dispersant solutions for each dispersant with concentrations ranging from 0.01 mg·mL^−1^ to 5 mg·mL^−1^ in 10 mL of DI at room temperature. We added 5 mg of graphite (0.5 mg·mL^−1^) to each vial. The solutions were ultrasonically sonicated for 10 min using a sonic dismembrator (Fisher Scientific, Hampton, NH, USA) Model 500 fitted with a 0.5 in diameter tip and set to 30 W. To prevent overheating of the solution during sonication, we simultaneously stirred the solutions with a magnet bar stir and surrounded them with an ice bath. The dispersions were centrifuged at 1000 rpm (150× *g*) for 10 min to remove unexfoliated graphite and multilayer graphene flakes. Due to the mismatch between water’s surface tension and solubility parameters (Table S3) and the ones for graphene, any un-exfoliated graphite and colloidally unstable graphene flakes would immediately precipitate at the bottom of the vial. We determined the graphene concentrations in the supernatants by measuring the absorbance using a UV/VIS/NIR spectrophotometer (PerkinElmer, Lambda 750, Waltham, MA, USA) in a quartz cell with a 1 cm path length at room temperature. The Beer–Lambert law was used to calculate the graphene concentration from the absorbance at 660 nm and a mass coefficient (Ꜫ) of 24.6 mL·mg^−1^·cm^−1^ [3,7,47]. We used the maximum graphene concentration achieved in the aqueous dispersions to estimate the appropriate dispersant concentrations for synthesizing graphene inks.

### 2.3. Developing Graphene Inks: Preparation and Formulation

Three different graphene inks, namely GGe, GTr, and GTw, were achieved (Figure 1) by using optimal quantities of dispersants, gelatin (0.1 mg·mL^−1^), triton X-100 (1.0 mg·mL^−1^), and tween-20 (1.5 mg·mL^−1^). The dispersant solutions were ultrasonicated with the graphene dispersion for 8 h using a sonic dismembrator (Fisher Scientific, USA), while an ice bath was used to prevent damage to the graphene flakes from the increase in heat. The excess dispersants were removed by centrifuging the dispersions for 2 h at 16,000 rpm (38,400× *g*) to precipitate (ppt) all carbon materials (colloidal stable and unstable graphene, unexfoliated graphite, carbon debris, etc.) to the bottom of the centrifuge tube. The supernatants were then carefully removed, and the resulting precipitates were further processed using sonication in 100 mL pure DI using the 500 W sonication probe for 1 h.

Two purification procedures were used to remove undesirable carbon materials. First, the graphene dispersions were centrifuged at 1 k rpm (150× *g*) for 30 min to remove unexfoliated graphite flakes and colloidal unstable graphene flakes. Next, the graphene supernatants were centrifuged at high speed for 2 h at 14 k rpm (29,400× *g*) to collect graphene flakes at the bottom of the vial, followed by dispersion in 100 mL pure DI using the 500 W sonication probe for 1 h.

The formulated aqueous-based graphene inks had a 2.2–2.5 cP viscosity and a surface tension of 30–35 dyne/cm at room temperature. The viscosity was measured using a viscometer (A&D, SV-10, Tokyo, Japan), and the surface tension was measured using a dynamic tensiometer (Dataphysics, DCAT11, Filderstadt, Germany). The graphene ink concentration was adjusted to 3 mg·mL^−1^ before the viscosity and surface tension were measured.

### 2.4. Characterization

#### 2.4.1. Characterization of Graphene Dispersions: Analyzing Stability, Morphology, and Composition

Zeta-potential (ZetaPlus/Bl-PALS, BrookHaven Instrument Corp., Holtsville, NY, USA) was utilized to validate the colloidal stability of the graphene dispersions. The dynamic light scattering (DLS) analysis was carried out at a wavelength of 633 nm utilizing a particle size analyzer (Zetasizer Nano S90, Malvern, UK) outfitted with a 4 mW laser and an avalanche photodiode detector (APD). Graphene dispersions were adjusted to 0.02 mg·mL^−1^ concentrations for DLS measurements.

Atomic force microscopy (AFM, Bruker, MultiMode8, Billerica, MA, USA) and transmission electron microscopy (TEM, JEOL JEM-2100F, Akishima, Japan) were used to analyze the morphology of graphene flakes. On the one hand, TEM is a powerful technique that provides essential information on the number of graphene layers and crystal structure, thanks to its high-resolution imaging at the atomic level [48,49]. On the other hand, AFM complements this by offering data on graphene’s thickness, roughness, and topography [50]. At room temperature, AFM images were collected in the tapping mode for samples prepared by drop-casting a drop of the diluted graphene solutions (0.1 mg·mL^−1^) on a freshly cleaned mica substrate and dried at 40 °C for 1 h. TEM samples were prepared by dipping lacey grids (TED PELLA, Redding, CA, USA) in the diluted dispersion (0.1 mg·mL^−1^). AFM and TEM samples were left to dry at room temperature overnight before the measurements.

The thermogravimetric analyzer calculated the polymer-to-graphene ratio and the composition temperature for graphene flakes (TGA, TA Instruments, TGA Q500, New Castle, DE, USA). The heating rate was 10 °C/min from room temperature to 1000 °C under air. Raman spectra were obtained from 400 cm^−1^ to 4000 cm^−1^ with a Raman microscope (Renishaw, inVia Reflex., Wotton-under-Edge, UK) at room temperature and a 532 nm excitation laser was used to determine the quality of graphene flakes and films. The characteristic Raman bands of carbon-based sp^2^ materials are about 1350, 1580, 2700, and 2900 cm^−1^ and are assigned to the D, G, 2D, and D′ bands, respectively. The D band is proportional to structural flaws and connected with the A_1g_ vibration mode of sp^2^ carbons, the G band with the E_2g_ phonon of sp^2^ carbon atoms, and the 2D band with a double resonance Raman process [7]. The peak intensity ratio (I_D_/I_G_) of the D and G bands is related to the average size of the sp^2^ domains and is used to measure the degree of order in graphene crystalline structures, termed the graphitization degree [7,51]. The UV/VIS/NIR spectrophotometer (PerkinElmer, Lambda 750, USA) was used to determine the optical bandgap using equations based on reflectance spectra [52]. Also, photoemission yield spectroscopy in the air (PYSA, Riken AC-2, Hitachi High Technologies, Tokyo, Japan) was used for valence band measurements. These analyses aided in drawing the graphene inks’ electronic structure as a function of the dispersant employed in the formulation of the inks.

#### 2.4.2. Comprehensive Characterization of Graphene Inks and AJP Films

The surface wettability of the printed films was assessed by measuring the contact angle of droplets of DI and graphene inks deposited on a cleaned PET substrate using a micro-pipette and analyzing the images with ImageJ software [53]. The smaller the contact angle obtained, the better the wettability achieved. Graphene films with varying printing layers were printed on top of the PET substrate using an AJP technique (Optomec, Albuquerque, NM, USA) with a 300 µm diameter nozzle and carrier gas/sheath gas rates set at 15 sccm/40 sccm. The stage was maintained at 70 °C to dry water during printing. The printed graphene films were incubated overnight at 40 °C to ensure water removal. The microstructures of the printed graphene films were examined by scanning electron microscopy (SEM, JEOL JCM-6000plus, Japan). The electrical resistance of the printed films was assessed using four-point probe resistance measurements (Lucas Labs 302, Thunder Bay, ON, Canada), while the thickness of the films was determined using profilometry (Bruker Dektakxt, USA). All measurements were taken at room temperature.

Graphene sensors were fabricated by aerosol jet printing graphene inks onto flexible screen-printed carbon electrodes on a PET substrate (Figure S2). The carbon electrodes were 10 cm long, 1 mm wide, and 5 µm thick, with 100 µm spacing between the carbon bars (Figure S2A). The graphene structures were 1 cm long, 400 µm wide, and 400 nm thick (Figure S2B). The printing gap was set at 300 μm, yielding a graphene printing width of 150 μm and a consistent spacing of 150 μm between adjacent graphene printings (Figure S2B). The electrical resistance of the as-fabricated sensors in air ambient was 200 kΩ as measured by a multimeter. The graphene-based sensors were exposed to different gases, including nitrogen (N_2_), hydrogen sulfide (H_2_S), and ammonia (NH_3_). The electrical resistance variation between every two carbon electrodes was measured using a programmed multimeter connected to a PC through an Arduino card [52,54]. The sensors’ response was given as normalized (R_gas_/R_air_), where R_air_ and R_gas_ are the electrical resistances of the sensors in air and gas, respectively, as a function of time upon exposure to the gases.

#### 2.4.3. Mechanical Flexibility Analysis of Graphene Films

This study investigated the mechanical flexibility of graphene films, which are carbon-topped electrodes similar to those used to fabricate graphene sensors (Figure S3). Bending tests were performed at 5° increments to determine the critical bending angles for the samples and bare carbon electrodes while measuring the resulting electrical resistance change. The electrical resistance of the printed graphene films (Figure S3A,B) was measured during bending. The mechanical flexibility of the samples was evaluated at their critical angles during repetitive bending cycles using a homemade setup (Figure S3D,E). This setup attached half of the sample to a glass slide, while the other half was connected to a moving glass slide (Figure S3C) linked to a linear motor (Velmex XSlide XN10, Bloomfield, NY, USA) that bent the sample at a rate of 6°/s. The electrical resistance of the samples was measured using a Keithley source meter (Keithley 2601A Sourcemeter, Cleveland, OH, USA) at a bias voltage of 6 V, and the results were reported as the normalized resistance (R/R_0_), where R_0_ and R represent the electrical resistance measured before and after bending the sample.

#### 2.4.4. Electrochemical Characterization of Graphene Inks

Electrochemical analysis techniques such as cyclic voltammetry (CV), square-wave voltammetry (SWV), and electrochemical impedance spectroscopy (EIS) were performed to investigate graphene’s electrochemical activity using a three-electrode Biologic SP-200 potentiostat (Bio-Logic Science Instruments SAS, France) with the EC-Lab software. The adsorption of dispersants on the surface of the graphene flakes can influence their electrochemical activity. CV and SWV were used to analyze the kinetics of the electrodes, while EIS was used to investigate the interfacial characteristics of the graphene layer [55]. The electrochemical cells were prepared by drop-casting 10 µL of a diluted graphene solution (0.125 mg·mL^−1^) on the active surface area of screen-printed carbon electrochemical electrodes (BioDevice Technology, Ltd., Nomi, Japan) with carbon working and counter electrodes and an Ag/AgCl reference electrode, as shown in Figure S4. After each graphene deposition, the cells were dried in an oven at 40 °C for 1 h. EIS, CV, and SWV were performed by placing a drop (20 µL) of Fe(CN)_6_^3−/4−^ electrolyte (5 mM K_3_[Fe(CN)_6_] and K_4_[Fe(CN)_6_] in 10 mM phosphate-buffered saline (PBS, pH = 7.4)) on the active area of an electrochemical cell. Blank measurements were taken for calibration before the graphene deposition. The CV measurements were carried out at a constant scan rate of 20 mV·s^−1^ from −400 V to 700 mV for 10 cycles, while the SWV curves were recorded from −100 mV to 600 mV. EIS was performed over a frequency range of 100 Hz to 200 kHz at an amplitude of 50 mV. The real and imaginary components of the complex impedance were denoted as Z_Re_ and Z_Im_, respectively. All experiments were conducted at room temperature.

Additionally, EIS was performed to investigate the conduction process of graphene nanostructures with gases using a two-electrode system (Hioki Chemical Impedance Analyzer, IM3590, Nagano, Japan). The EIS for graphene gas sensors was investigated with exposure to N_2_ (inert), H_2_S (acidic and electron-withdrawing molecule), and NH_3_ (basic and electron-donating molecule) gases. The measurements were taken after a 1 h pause between gas insertions, and sensor recovery was evaluated by exposing the sensors to room-temperature air after opening the chamber’s lid.

## 3. Results and Discussion

This study aims to synthesize graphene inks by exfoliating graphene directly from raw graphite into the water, which is considered the most environmentally friendly solvent. However, graphene is not readily dispersible in water due to the significant differences in their respective HSPs and surface tension (Table S3). To address this, surface-active chemicals or dispersants were added to balance the surface energy between the graphene and the solvent. This study used amphiphilic dispersants such as gelatin, triton X-100, and tween-20 to stabilize graphene in the aqueous solution. These dispersants consist of hydrophilic (polar) and hydrophobic (non-polar) components, allowing them to immobilize on the surface of the graphene through hydrophobic interactions while the polar parts remain free in the aqueous medium [56,57]. This leads to a hairy layer surrounding the graphene layers, providing steric stabilization. Triton X-100 and tween-20 were used to stabilize the graphene flakes sterically [32], whereas gelatin, which contains both ionic and nonionic components, likely employs both steric and static stabilizing mechanisms [58]. These dispersants achieve a stable graphene dispersion by adsorbing onto the surface of graphene, which is critical for successfully formulating graphene inks [59].

**Figure 2:**
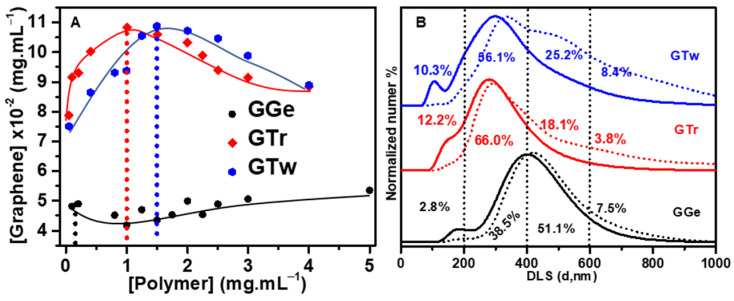
(**A**) Relationship between dispersant concentration and graphene concentration in aqueous dispersions. (**B**) Diameter distribution analysis of graphene flakes in GGe, GTr, and GTw dispersions using DLS. The dotted and solid curves show the flake size distribution after the first (1 k rpm) and second (14 k rpm) purification steps, respectively. The percentages inside the figure represent the flake size distribution for the final ink formulations after the second (14 k rpm) purification step.

The concentration of dispersant used and the surface coverage of the graphene flakes are key factors in achieving stable graphene dispersions with high concentration and good electrical performance [7,51]. Therefore, it is important to optimize the dispersant content. In this study, we started with a 0.5 mg·mL^−1^ graphite concentration in 10 mL DI water and measured the optical absorbance at 660 nm to determine the concentration of exfoliated graphene. Figure 2A shows that increasing triton X-100 and tween-20 concentrations gradually up to 1 mg·mL^−1^ and 1.5 mg·mL^−1^ led to a maximum graphene concentration of 0.11 mg·mL^−1^, representing 20% of the initial graphite mass successfully exfoliated. The rest (80%) remained unexfoliated at the bottom of the dispersion. Triton X-100 required 1 mg·mL^−1^ to achieve the highest graphene concentration, while tween-20 needed 50% more (1.5 mg·mL^−1^) to achieve the same concentration, suggesting that triton X-100 is more effective at adsorbing on the surface of graphene than tween-20. Above 1 mg·mL^−1^ and 1.5 mg·mL^−1^ for triton X-100 and tween-20, respectively, the graphene concentration decreased due to the depletion flocculation mechanism [7,51]. In comparison, increasing the gelatin concentration from 0.1 mg·mL^−1^ to 5 mg·mL^−1^ had no discernible effect on increasing the graphene concentration beyond 0.05 mg·mL^−1^ (2.2 times less than the dispersion with triton X-100 and tween-20). We assumed that the graphene surface was saturated at the optimal dispersant concentrations, which optimized the size of the potential barrier surrounding the flakes for the best stabilization [7,51]. Therefore, we used appropriate dispersant concentrations of 0.1 mg·mL^−1^, 1 mg·mL^−1^, and 1.5 mg·mL^−1^ for gelatin, triton X-100, and tween-20, respectively, to prepare the graphene inks denoted as GGe, GTr, and GTw. Notably, the dispersant concentrations used in this study were considerably lower than those applied in developing graphene ink (>10 mg·mL^−1^) [60].

Graphene in aqueous solutions develops a zeta-potential, which refers to the potential and electric double layer formed at the interface and is highly associated with dispersion stability [61]. A high magnitude zeta-potential indicates repulsion between flakes and can imply stable dispersions. For diluted dispersions (0.02 mg·mL^−1^) at pH 6.4, the zeta-potential for GTr and GTw was found to be −38.5 mV, while for GGe it was −41.5 mV. The greater zeta-potential for GGe can be attributed to the contribution of both steric and static stabilizing mechanisms. The negative zeta-potential indicates that the net surface charge of the graphene flakes is negative, and the zeta-potential values are sufficient to prevent graphene flakes from precipitating, ensuring stable dispersions.

DLS and TGA techniques were employed to better engineer the graphene dispersions’ qualities in order to evaluate the effect of the dispersants’ properties and purification steps involving centrifugation at 1 k and 14 k rpm. Controlling the flake size distribution is crucial to avoid nozzle clogging and complications during printing. Additionally, the dispersant-to-graphene ratio, which determines the surface coverage of graphene flakes by dispersants, is critical for optimizing charge transport in graphene films [7], and flake size distribution [62].

DLS was used to assess the flake size distribution of the inks as a large-scale evaluation of the mean flake size in the graphene dispersion. After the first purification stage involving centrifugation at 1 k rpm for 30 min, graphene dispersions exhibited a diameter distribution range of 50 nm to 800 nm (Table 1, Figure 2B), with over 90% of flakes ranging from 50 nm to 600 nm, consistent with the flake size produced by the LPE process [63]. The peak maxima in the DLS curve distributions for GTr and GGe based on particle number were centered between 300 and 500 nm, while the GTw DLS curve distribution was centered between 200 and 400 nm. According to the area under the curve, GTr and GGe dispersions contained 46.2% and 48% of flakes smaller than 400 nm (<400 nm), respectively. The GTw dispersion contained 67.8% flakes < 400 nm, indicating that it contained 20% smaller flakes than the GTr and GGe dispersions. Flakes larger than 400 nm (>400 nm) accounted for 52%, 53.8%, and 32.2% of GGe, GTr, and GTw, respectively.

After the second purification process (centrifugation at 14 k rpm for 2 h), the flake size distribution of the final graphene dispersions obtained had shrunk to 100–800 nm, and significant changes were observed in the flake distribution for each sample, as outlined in Table 1 and illustrated in Figure 2B. The DLS curves showed a decrease in the average flake size for GTr and GTw, with a peak in the 200–400 nm range. The flake size distribution observed in the initial DLS curve for GGe was retained, which was centered in the 300–500 nm range. Specifically, a higher percentage of smaller flakes (<400 nm) was found for GTr and GTw, with 72.2% and 66.4%, respectively, compared to GGe, which had only 41.3% flakes < 400 nm. Furthermore, a more significant proportion of larger flakes (>400 nm) was observed for GGe compared to GTr and GTw.

These results suggest that the type of dispersant used can influence the flake size distribution. It was also observed that the second purification process effectively eliminated graphene flakes > 400 nm from the GTr and GTw inks, resulting in more symmetrical DLS curves. These findings highlight the importance of carefully controlling the dispersion and purification of graphene to optimize its properties for various applications, particularly as nanofillers in printed films, where smaller flakes can improve charge transport and increase electrical conductivity.

**Figure 3:**
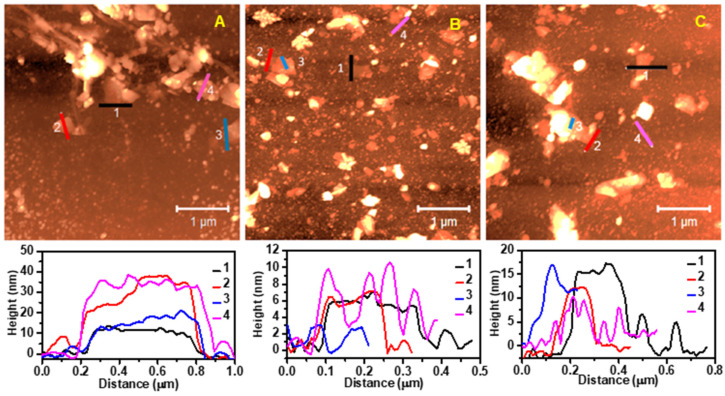
AFM images and corresponding surface profiles of graphene nanosheets from (**A**) GGe, (**B**) GTr, and (**C**) GTw. The surface profiles illustrate the thickness variation caused by the coating of adsorbed dispersant-like residue on both the graphene surface and the mica substrate.

**Figure 4:**
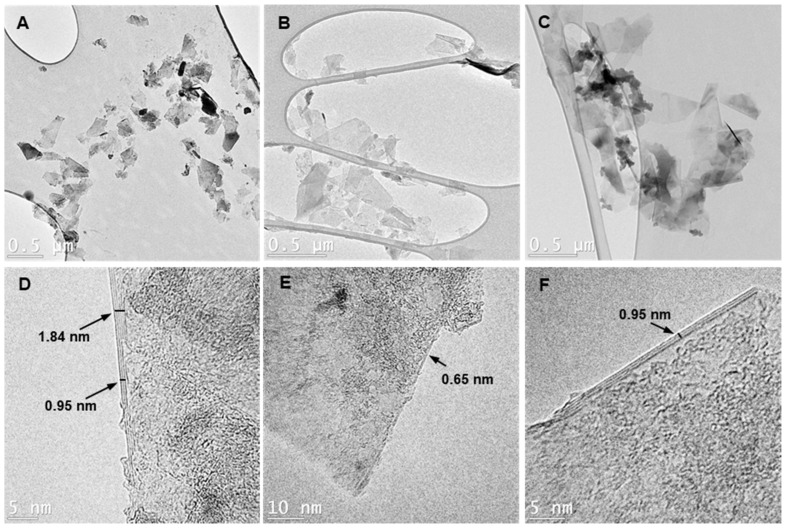
TEM images showing the morphology of graphene sheets obtained from (**A**,**D**) GGe, (**B**,**E**) GTr, and (**C**,**F**) GTw samples. The low-contrast images (**A**–**C**) confirm the presence of 2D graphene sheets in all three samples, while the high-contrast images (**D**–**F**) reveal differences in the number of layers and surface features.

The morphological properties of the graphene samples were investigated using AFM and TEM, as shown in Figure 3 and Figure 4, respectively. AFM imaging revealed that the size distribution of the exfoliated nanosheets in the three graphene inks (GTr, GTw, and GGe) ranged from 200 nm to 600 nm, consistent with the major flake dispersion reported in DLS. The thickness of the exfoliated nanosheets estimated from the surface profile varied from 6 nm in GTr to a few tens of nanometers in GTw and GGe, indicating that the LPE generated multilayer graphene flakes in the aqueous inks.

One consistent observation in the AFM images was the presence of a coating of deposited dispersant-like residue on the graphene and mica substrate surfaces. These coatings achieved a height of 10 nm on top of the mica substrate with GTr (Figure 3B, profile 4) and GTw (Figure 3C, profile 4), and a height of 25 nm with GGe (Figure S5). Additionally, the dispersant accumulations on the graphene’s surface reached a height of 2 nm for GTr (Figure 3B, profile 3), 5–10 nm for GTw (Figure 3C, profile 3), and 10–20 nm for GGe (Figure 3A, profile 2). Due to these accumulations on the mica and everywhere around graphene flakes (bottom and top), estimating the number of graphene layers by measuring the thickness of exfoliated graphene nanosheets is challenging.

The TEM images in Figure 4 and Figure S6 support our earlier observations. The low contrast in Figure 4A–C confirm that the 2D graphene sheets in our sample are exfoliated and dispersed in an aqueous medium. These sheets have dimensions of 400–600 nm in length and 150–300 nm in width. However, TEM micrographs also revealed that the individual graphene flakes tend to re-stack as the solvent dries, forming overlapping graphene sheet accumulations. The dark-light patches in high-contrast TEM images (Figure 4D–F) are likely the layered dispersant adsorbed on the graphene surface.

Our TEM images showed that the GGe sample had large dark areas identified as piled gelatin (Figure S6A,B). The TEM images also showed that the GGe sheets were multilayered, with thicknesses ranging from 1.15 nm to 4.1 nm in different locations within a flake. This indicates uneven graphene exfoliation by gelatin, resulting in 4 to 12 layers per sheet, as shown in Figure 4D and Figure S6C. In contrast, the TEM images confirmed that GTr (Figure 4E and Figure S6E–H) and GTw (Figure 4F and Figure S6I–L) formed only a few layers of graphene sheets, with thicknesses ranging from 0.61 nm to 1.53 nm, corresponding to 2 to 5 graphene layers per sheet.

**Figure 5:**
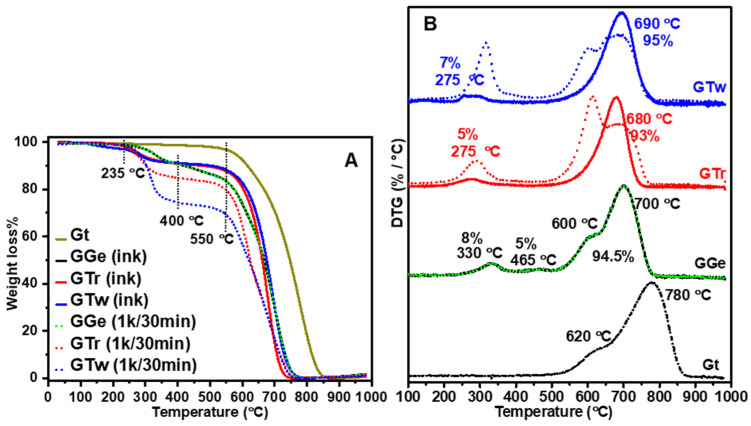
(**A**) TGA and (**B**) DTG thermograms of raw Gt, GGe, GTr, and GTw. The dotted and solid curves represent the decomposition curves after the first (1 k rpm) and second (14 k rpm) purification steps, respectively. The percentages inside the figure indicate the quantities of graphene and dispersants for the final ink formulations after the second purification step.

To evaluate the impact of purification on the dispersant-to-graphene ratio and graphene’s thermal characteristics, we conducted TGA and DTG analyses. As shown in Figure 5A, raw Gt (7–10 m) decomposed over a broad range of temperatures (550–850 °C), with noticeable DTG peaks at 780 °C and a shoulder at 620 °C (Figure 5B). After a 1 k rpm centrifugation procedure, the obtained graphene flakes from GTr and GTw showed a breakdown range of 500–750 °C, while GGe exhibited a substantially higher decomposition temperature range of 500–780 °C (Figure 5A). We calculated the quantities of dispersants (Table 2) as 14% for gelatin (250–580 °C) in GGe (Figure S7), 19% for triton X-100 (200–400 °C) in GTr, and 26% for tween-20 (200–450 °C) in GTw, corresponding to the initial dispersant concentration used during ink synthesis. The DTG (Figure 5B) revealed broad peak structures for mass loss in the 500–780 °C range that could be deconvoluted into sub-peaks, indicating the diverse graphene flakes synthesized from the inks. However, further research is needed to understand these results better.

The final inks (shown in Figure 5A,B) underwent a significant decrease in polymer content for triton X-100 and tween-20, with GTr and GTw containing only 5% and 7% polymer, respectively, after the second purification phase (14 k rpm) (as detailed in Table 2). This suggests that the removed triton X-100 and tween-20 dispersants are primarily bound to graphene flakes larger than 400 nm, which were eliminated along with their supernatants during the 14 k rpm centrifugation step. Additionally, the DTG peaks for GTr and GTw improved to semi-symmetrical shapes at 680 °C and 690 °C, respectively, indicating that eliminating larger graphene flakes was associated with the flakes’ thermal degradation. Meanwhile, the DTG curve for GGe showed a slight decrease in gelatin content from 14% to 13% (Figure 5B and Table 2). This implies that the excess gelatin dispersion was successfully removed during the precipitation step of centrifugation at 16 k. The thermogram for GGe maintained a comparable DTG (Figure 5B), suggesting only a minor change in flake size distribution as determined by DLS analysis.

**Figure 6:**
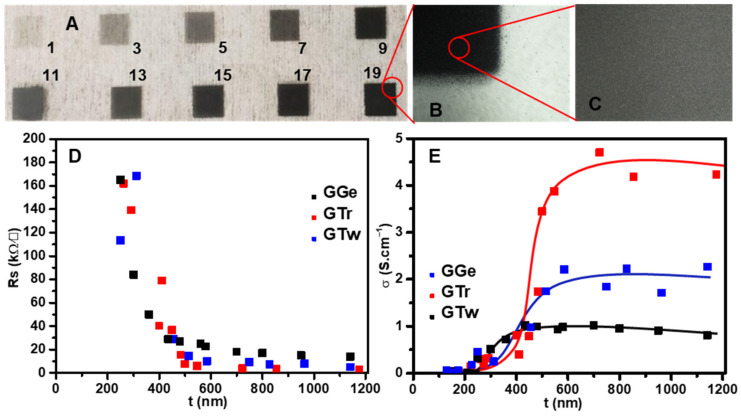
(**A**) Optical microscope images showing AJP GTr films on a PET substrate at different printing pass numbers. (**B**,**C**) Optical microscope images depicting the center and edge of a graphene film. (**D**) Rs and (**E**) σ plotted against the thickness (t) of the films.

The two-step purification process significantly improved the flake size distribution and dispersant-to-graphene ratio, resulting in better graphene printability by removing large graphene flakes and graphene ruptures created during the sonication process. Thus, we transformed the graphene dispersions into graphene inks (GGe, GTr, and GTw) at concentrations of 3 mg·mL^−1^, which remained stable with excellent dispersity even after being stored for over a month. Using an AJP, we deposited the graphene inks onto a flexible PET substrate, producing thin films (Figure 6A–C) after a given number of printing passes.

The inks flowed smoothly through the nozzle during printing without requiring additional modifications to control their properties, such as viscosity, surface tension, printing flow, or rheology modifiers. To assess their performance characteristics, we evaluated the printability, wettability, and electrical behavior of the graphene inks as a function of the type of dispersant used. These inks appear to have promising potential considering the two essential performance characteristics that ink should have: good printability and printing definition.

The films’ thicknesses increased as the printing passes increased, with an average layer thickness of ≈32 nm. Figure 6A displays the graphene films produced by AJP GTr ink on a PET substrate with varying printing passes, while Figure 6B,C show high-magnification images depicting uniform film structures with well-defined edge patterns.

In terms of the electrical behavior, thicker films had lower sheet resistance (Rs), settling at 7.5 kΩ/□, 3.6 kΩ/□, and 2.6 kΩ/□ for GTr films at 500 nm, 800 nm, and 1.15 µm, respectively. For GTw films, Rs settled at 14.4 kΩ/□, 7 kΩ/□, and 5 kΩ/□ for thicknesses of 500 nm, 800 nm, and 1.15 µm, respectively, which is approximately double the Rs recorded for GTr films with the same thicknesses. GGe films had the highest sheet resistance, with 27 kΩ/□, 17 kΩ/□, and 14 kΩ/□ for film thicknesses of 500 nm, 800 nm, and 1.15 µm, respectively. The conductivity (σ) was calculated using the reciprocal of resistivity (ρ) (σ = ρ^−1^), where ρ was calculated from the multiplication of Rs and t [64]. σ was steady for t > 500 nm (Figure 6E), averaging 4.5 S·cm^−1^, 2 S·cm^−1^, and 1 S·cm^−1^ for GTr, GTw, and GGe, respectively.

The electrical conductivity of the printed graphene films, as shown in Figure 6E, is lower than the theoretical value of 106 S·cm^−1^ for a single graphene sheet. This suggests that the resistance of the inter-flake junction dominates in the graphene films [18,65]. Post-treatments can be employed to enhance the interlayer connection of the graphene, improving the conductivity of the printed graphene films. Although the conductive properties of graphene inks (GTr, GTw, and GGe) are not equivalent to those of metallic inks, they are still sufficient for fabricating chemiresistive flexible sensors.

Other researchers have previously reported conductivities for graphene inks ranging from 1 to 10^5^ S·cm^−1^ [3,13,16,60]. These inks require complicated and costly preparation stages, such as high-temperature calcination [3,16,60], and acid treatment [16]. Additionally, using toxic solvents like NMP and terpineol in some of these inks makes them unsuitable for flexible substrates in printed and flexible electronic fabrication [13]. Moreover, high dispersant concentrations (>10 mg·mL^−1^) like ethyl cellulose (EC) were required in these inks, which can create nanofillers of conjugated structures between graphene flakes, improving charge transmission through the film when calcined at temperatures above 300 °C [3,60].

In contrast, our inks surpass these earlier graphene inks due to the simplicity of our technique and the low dispersant concentrations (0.1–1.5 mg·mL^−1^) used in the ink formulation. Our inks were printed without any supplementary additives to regulate printability on the PET substrate and employed a moderate drying temperature of 40 °C, achieving good conductivity in the range of 1–4.5 S·cm^−1^. Our inks are suitable for flexible substrates in printed and flexible electronic fabrication, and their simple and cost-effective preparation stages make them ideal for large-scale manufacturing.

**Figure 7:**
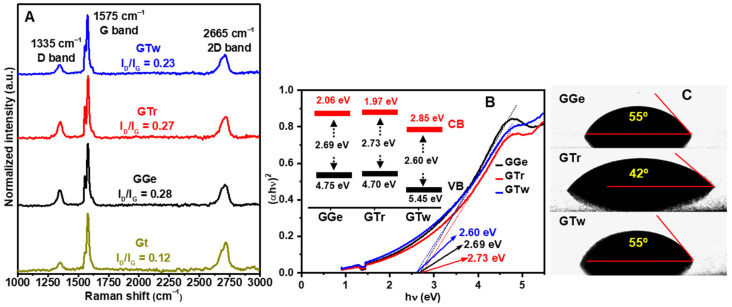
(**A**) Raman spectra of printed films of graphene inks (GGe, GTr, and GTw) versus raw Gt. (**B**) Optical absorption spectra of graphene inks plotted as (F(R)vh)2 versus energy (vh). (Inset) Band structure diagram of the graphene inks, where VB and CB represent the valence and conduction bands, respectively. (**C**) Contact angle measurements on PET substrates for GGe, GTr, and GTw graphene inks, indicating their wetting capability and adhesion properties.

The electrical properties of printed graphene structures are primarily determined by the quality of the materials and the interconnectivity between flakes [7,18,65]. Raman spectroscopy is commonly used to investigate the quality of the sp^2^ structure (or graphitization degree), which measures the intensity ratio between D and G peaks (I_D_/I_G_ ratio). Figure 7A illustrates distinctive peaks for raw graphite and graphene inks at 1335 cm^−1^ (D band), 1574 cm^−1^ (G band), and 2666 cm^−1^ (2D band). The displayed spectra show the median representations of Raman spectra collected at ten different locations on each sample. The raw graphite Raman spectrum exhibits an I_D_/I_G_ ratio of 0.12, indicating the feature of well-crystallized bulk graphite. Exfoliation results in a minor increase in the D band, leading to a slight rise in I_D_/I_G_ ratios (0.23–0.28), consistent with the values found for graphene inks (I_D_/I_G_ ≈ 0.17–0.45) [3,13,16,60]. These results suggest that the exfoliation process causes a slight increase in the disorder level of the graphene layers compared to raw graphite.

The bandgap and valence band measurements were performed to assess the quality of the graphene π-conjugated system in the inks, as shown in Figure 7B. The valence band and bandgap values for the graphene inks ranged from 4.7 to 5.45 eV and 2.60 to 2.73 eV, respectively. These values are consistent with those observed in high-quality conjugated graphene structures [66,67,68,69]. However, while Raman spectroscopy and bandgap/valence band analysis provide valuable insights into the electronic properties of graphene inks, they do not fully explain the changes in the electrical characteristics that result from alterations in the graphene sp^2^ structure induced by dispersants.

The flake interconnectivity and the wettability of the inks significantly influence the electrical conductivity of the graphene films. The interaction between the substrate and the graphene inks is governed by the match between the inks’ surface tension and the surface energy of the PET substrate (≈45 mJ·m^−2^) [70,71], which is a crucial factor affecting the printing quality and film smoothness, both of which impact the electrical conductivity of the graphene films. On the other hand, the wetting capability of the inks is a critical parameter that determines their adhesion to the substrate. The contact angle of DI on the PET substrate was measured to be 78°, which is consistent with values seen in the literature [72]. The droplet shape of the graphene inks immediately after dropping on the PET substrate is shown in Figure 7C. Contact angle measurements revealed that GTr had the smallest contact angle of 42°, indicating the best wetting capability and adhesion over the PET substrate, while GTw and GGe had a contact angle of 55°, indicating a relatively lower adhesion property than GTr ink.

**Figure 8:**
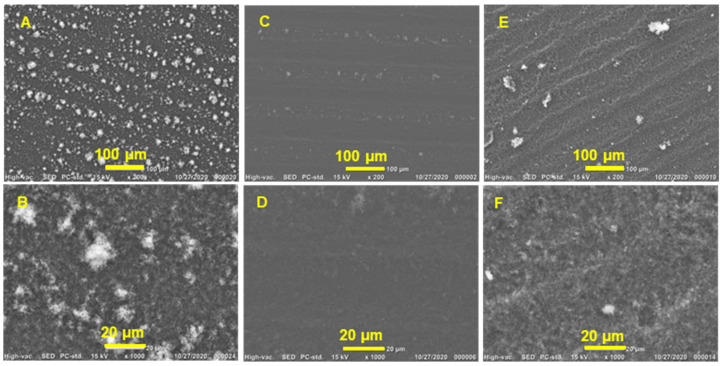
SEM images of graphene films printed on PET substrate from (**A**,**B**) GGe, (**C**,**D**) GTr, and (**E**,**F**) GTw, depicting the morphology of the film’s architecture.

The interconnectivity of graphene flakes is crucial for promoting charge transport within graphene films and increasing electrical conductivity, and it works in collaboration with the properties of the individual flakes. The surface morphology of the graphene films produced on PET substrates was analyzed using SEM. Figure 8 shows a surface view of the graphene film, demonstrating how the film’s architecture is made up of stacked flakes with different levels of agglomeration and wrinkle structures. Thickly coated GGe films (13% gelatin content and 12 µM CMC) [73] exhibited white accumulations and a highly wrinkled structure (Figure 8A,B), likely due to the formation of micelles during solvent drying, which disrupted the interlayer connections between graphene flakes and decreased the electrical conductivity of the GGe film. In contrast, GTw films (7% tween content and 42 µM CMC) [74] had a lower degree of wrinkling and accumulation (Figure 8E,F), which can be attributed to the higher CMC that reduced the formation of micelles. The GTr film (5% triton X-100 content and 200 µM CMC) [74] had a densely packed structure and a smooth film surface (Figure 8C,D) due to its high CMC that reduced the formation of the micelles. This reveals good interconnectivity between adjacent graphene nanosheets, enhancing electrical conductivity. High-magnification SEM images confirmed that the GTr film had a well-stacked structure with good contact between adjacent graphene flakes, while GTw and GGe films showed more agglomeration and less interconnectivity.

The DLS analysis revealed a high percentage (12.2%) of GTr flakes < 200 nm, which could enhance the electrical conductivity of the graphene films. Small flakes can fill in the gaps in the network structures, increasing the flake interconnectivity and the uniformity and density of the printed films. This is because they act as a bridge between graphene flakes by providing network frames and π-π interactions with the graphene flakes, creating additional charge transport pathways within the film. Overall, GTr has superior electrical conductivity (4.5 S·cm^−1^), which can be related to the high percentage of nanofillers of <200 nm flake size (12.2%), a low dispersant-to-graphene ratio (5%), good quality (I_D_/I_G_ ≈ 0.27), good wettability (θ ≈ 42°) on PET, and a smooth and uniform film on PET.

**Figure 9:**
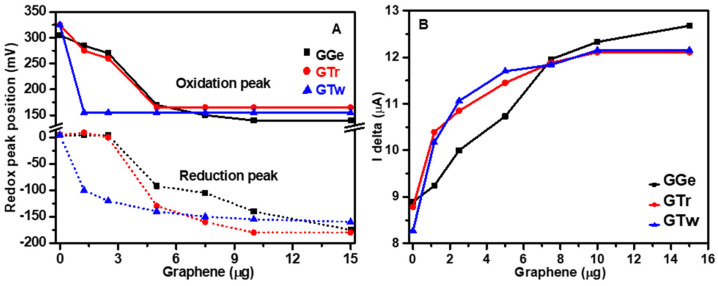
Electrochemical analysis of graphene inks (GGe, GTr, and GTw) deposited on screen-printed carbon electrochemical electrodes. (**A**) The shift in redox peaks position and (**B**) Current height in SWV curves.

Electrochemical studies were carried out to investigate the impact of dispersants on the microscopic nanostructure of graphene inks and their charge transfer properties. As a control, CV analysis of bare electrochemical cells demonstrated distinct, well-defined redox peaks, indicating that the cell triggers electron transfer processes without needing graphene deposition (Figure S8A–C). Following graphene deposition on the electrochemical cell’s active surface area, the CV curve properties (redox peak position, potential difference, and curve shape) varied among the graphene samples (GGe, GTr, GTw), implying that the dispersants influence the inks’ charge transfer process. After graphene deposition, the electrochemical cells immediately and progressively shifted the oxidation peak to a lower voltage, stabilizing around 140–165 mV. Although the final redox peaks for graphene inks nearly converged, the dispersants’ shift mechanisms differed. Tween-20 showed a relatively prompt shift in the redox peaks (Figure 9A), while Triton X-100 and gelatin dispersants aided a slow and relatively extended mechanism shift (Figure 9A). The rapid settlement of redox peaks in GTw suggests more persistent redox reactions at the graphene surface, resulting in more consecutive reactions than in GTr and GGe.

GGe exhibited greater defined redox peaks than GTr and GTw, and the redox peaks for GGe were of higher intensity than the bare electrochemical cell. This finding is consistent with previous reports demonstrating that gelatin enhances proton diffusion due to the presence of both ionic and nonionic moieties attributed to its chemical structure [28,29]. In contrast, the CV curves for GTr and GTw were broader than those for the bare electrochemical cell, which could be attributed to slower electron transfer kinetics and reduced reaction reversibility [55]. This suggests that GTr and GTw films act as a barrier to negatively charged species, resulting in electrostatic repulsions between graphene nanoflakes and the negatively charged analytes, increasing cell polarization, and delaying the rate of the redox reaction for GTr and GTw.

Figure S8D–F show the results of the SWV analysis conducted on bare electrochemical cells before and after loading with graphene inks. The bare cells exhibited broad voltammograms with peak currents ranging from 8.27 µA to 8.89 µA. Upon loading with graphene inks, a progressive increase in the peak current was observed, with a shift towards a lower voltage step, especially for GTr and GTw. For instance, loading with 1.25 µg of GTr led to an increase in the peak current to 10.39 µA (+15.5%), which further increased to a peak current saturation of 12.11 µA (+27.5%) at a 10 µg mass load (Figure 9B and Figure S8E). Similarly, loading with 1.25 µg of GTw resulted in a peak current of 10.17 µA (+18.7%), which reached a saturation current of 12.15 µA (+31.9%) at a 10 µg load (Figure 9B and Figure S8F). GGe showed a small rise in the peak current of 9.24 µA (+3.8%) at a 1.25 µg load, which increased to 12.33 µA (+27.9%) at a 10 µg load (Figure 9B and Figure S8D). The rapid rise in peak current for GTr and GTw indicates an improvement in the transport of electroactive chemicals after graphene deposition, which was facilitated by the nonionic dispersants: triton X-100 and tween-20. Subsequently, SWV revealed a shift to a lower potential with graphene deposition, with GTw demonstrating a more stable complex between graphene and Fe(CN)_6_^3−/4−^.

EIS is a powerful technique that is widely recognized for its ability to capture intricate and complex electrical changes occurring on the surface of various materials. This includes the grain bulk, grain boundary, and interface between the electrode and sensor thin film, resulting from their interaction with analytes such as ionic solutions or gases [75]. Nyquist plots typically show two semicircles when examining bare electrochemical electrodes (without graphene), as Figure S8G–I illustrate. The semicircle at high frequency represents the electrode’s internal resistance and the electrode-graphene contact’s resistance [76,77,78]. Meanwhile, the semicircle at low frequencies is related to the resistance for charge transfer through the electrode–graphene film contact (diffusion resistance of the electrolyte ions) [76,77,78].

In contrast, following the deposition of graphene nanoflakes such as GTr, GTw, and GGe, the maximum bulk electrolyte resistance of a non-treated screen-printed electrochemical electrode remained at 3 kΩ, indicating that the porous morphology of the deposited graphene nanoflakes provides an accessible surface for electrolyte ions to penetrate deeply into the nanopores. This results in low charge resistance [76,77,78]. Alternatively, the low-frequency semicircle gradually shrinks when graphene inks are drop-casted, reducing electrode resistance for charge transfer events at the donor/acceptor interfaces. This indicates a transition from a capacitive to a resistive response.

**Figure 10:**
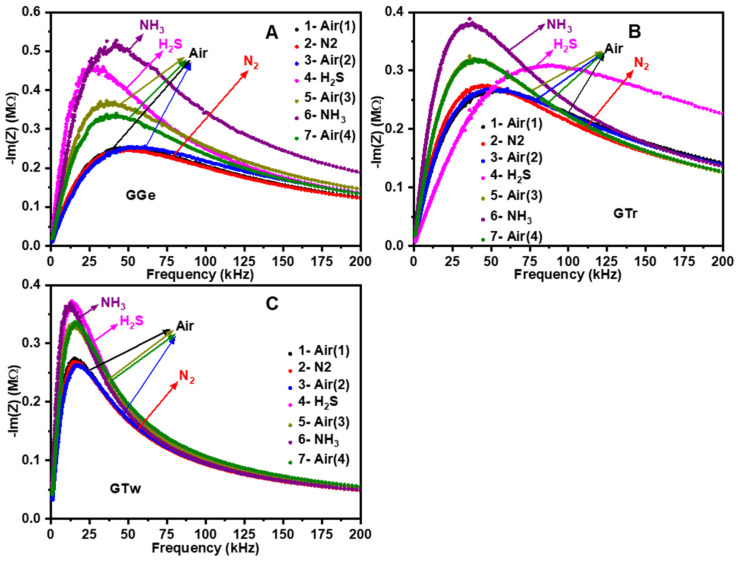
Impedance plot of printed graphene sensors (**A**) GGe, (**B**) GTr, and (**C**) GTw in gas environments (N_2_, H_2_S, NH_3_).

The investigation of the conduction process in the dry-solid state involved exposing printed graphene sensors (Figure 10) to N_2_, H_2_S, and NH_3_ gases. The response of the sensors to these gases at room temperature and 30% relative humidity is shown in Figure S9. Graphene’s response to gases can be determined by analyzing the EIS frequency regime in the Nyquist plot, which can reveal the material’s active structures and the target gas’s location, whether on the grain bulk, grain boundaries, or the metal/electrode contact. The sensing thin film to electrode contact can be attributed to the low frequency, while the contribution of grain boundaries and bulk can be observed at mid and high frequencies, respectively [79,80]. Each EIS figure displays seven curves, representing the sensor responses to gases in the following sequence: in the air as the base gas before exposure to the target gases (Air 1 and Air 3), after exposure to the target gas (N_2_, H_2_S, NH_3_), and after opening the chamber’s lid to expose the graphene sensor to the air again (Air 2 and Air 4).

In the air (Air 1), the sensors (GGe, GTr, GTw) have an initial resistance of 200–300 kΩ. However, the graphene sensors’ Nyquist plot showed no change after being exposed to the inert gas (N_2_), indicating that there is no charge transfer from graphene to nitrogen (Figure S9A and Figure 10A–C). Later, the chamber was opened to neutralize the sensors with air (Air 2). Upon exposure to H_2_S, an acidic and electron-withdrawing gas, the GGe-based sensor (Figure S9B and Figure 10A) and GTw-based sensor (Figure S9B and Figure 10C) exhibited almost constant resistance at high frequencies, while the GTr-based sensor showed an increase in resistance at mid and low frequencies. This indicates that graphene in GGe and GTw has low electrochemical catalytic activity towards H_2_S gas molecules and that electron transfer occurs mainly from graphene boundaries to H_2_S molecules. In contrast, the Nyquist plot for GTr (Figure S9B and Figure 10B) expanded and shifted to a higher frequency, indicating a reduction in electron density due to the withdrawal of electrons from a greater surface area of the graphene (boundaries and bulk). After opening the chamber’s lid (Air 3), the Nyquist plot was restored, showing increased electron density for graphene structures. This indicates that trapped electrons from adsorbed H_2_S molecules were reversibly released into the graphene.

Interestingly, the graphene sensors showed an unexpected sensing response when exposed to the basic and electron-donating gas (NH_3_). It was anticipated that NH_3_ molecules would donate electrons to graphene flakes, increasing the matrix electron density and minimizing resistance. However, the sensors’ response can be explained by forming acidic NH_4_^+^ ions via dissociating of NH_3_•H_2_O [81]. The adsorption of NH_4_^+^ ions can alter the exterior conduction properties of graphene interfaces [82], decreasing electron density and increasing resistance.

In contrast, the GTr-based sensor (Figure S9C and Figure 10B) and GTw-based sensor (Figure S9C and Figure 10C) showed increased resistance at mid and low frequencies while retaining resistance at high frequencies. This suggests that electron transport occurs mainly between graphene boundaries and NH_4_^+^ molecules. The Nyquist curve for the GGe-based sensor (Figure S9C and Figure 10A) expanded at high frequency, illustrating the loss of electron density due to electron withdrawal from graphene boundaries and bulk. The Nyquist pattern was restored after the sensors were exposed to air again (Air 4) by withdrawing electrons from NH_4_^+^ molecules. Further investigations are needed to understand these phenomena fully. However, the findings demonstrate that dispersants can influence the reactive component of graphene (bulk and boundaries), resulting in electrical activation towards contemporary gases.

**Figure 11:**
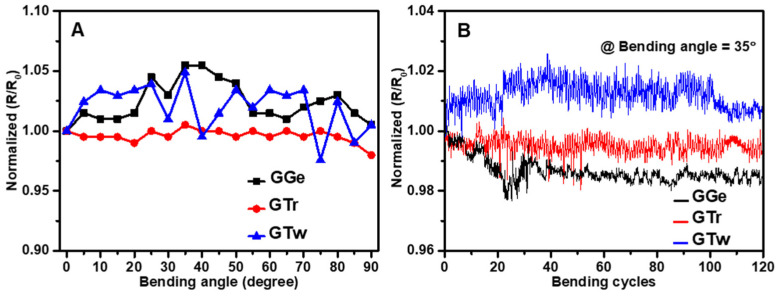
(**A**) Normalized resistance of AJP graphene films as a function of bending angle. (**B**) Variation in normalized resistance of printed graphene films under repetitive bending at a 35° bending angle.

Wearable printed devices with flexibility are highly desirable for their potential use in IoT applications. The critical angle at which the films fail electrically was determined to assess the mechanical flexibility of AJP graphene films. Results showed that GGe and GTw films had a normalized R/R_0_ ratio increase of 5% against bending angles up to 90° (Figure 11A). On the other hand, GTr exhibited robust mechanical behavior, indicating strong adhesion to the carbon electrodes and correlating with SEM and contact angle observations. Previous studies have validated the mechanical flexibility of carbon electrodes by demonstrating a minimal change in resistance when bent to 90° without losing conductivity [83]. The increase in resistance observed in the present study is due to a change in the orientation and distance between graphene flakes in the printed films. The flexibility of the sensors was then assessed based on their ability to withstand repeated bending at the predetermined critical angle. Results showed no significant change in resistance (2% fluctuation) in any of the printed graphene films after 120 bending cycles at a bending angle of 35° (bending radius 1 cm) (Figure 11B). These findings are consistent with other printed graphene nanocomposites that have demonstrated high durability (>1000 bending cycles) at bending radii ranging from 1 mm to 1 cm [84,85,86]. These results suggest that the formulated graphene films have good mechanical flexibility and could be promising for wearable and flexible IoT devices.

## 4. Conclusions

This study developed practical water-based graphene inks. The inks were formulated with low dispersant concentrations (0.1–1.5 mg·mL^−1^) and printed on PET substrates using an AJP without additional additives for printability. The drying temperature was kept moderate (40 °C), and the resulting inks demonstrated good conductivity (1–4.5 S·cm^−1^) suitable for chemiresistive sensor development. The study revealed that the dispersant used in the inks significantly influenced the graphene concentration achieved in the dispersion, the flake size distribution, number of graphene layers per flake, and the dispersant-to-graphene ratio, which in turn affected the inks’ wettability and smoothness on the substrate.

Triton X-100 was found to be the most effective dispersant for formulating the graphene ink (GTr) due to its ability to produce stable aqueous ink with low-defect few-layer graphene, high production yield efficiency, the highest percentage of graphene flakes < 200 nm, good printability, and quality of printed films, as well as good electrical conductivity (4.5 S·cm^−1^) and mechanical flexibility. The results suggest that GTr is suitable for fabricating chemiresistive sensors for IoT healthcare and environmental applications due to its appealing properties and non-toxic ingredients.

## Figures and Tables

**Table 1:** Graphene flake size distribution measured by DLS at first 1 k rpm and secondly 14 k rpm purification steps.

	GGe		GTr		GTw	
d (nm)	1 k	14 k	1 k	14 k	1 k	14 k
<200	6.3	2.8	6.1	12.2	3.1	10.3
200–400	41.7	38.5	40.1	66.0	64.7	56.1
400–600	42.2	51.1	45.5	18.1	29.8	25.2
>600	9.8	7.5	8.3	3.8	2.4	8.4

**Table 2:** Dispersant quantities bound to graphene, as measured by TGA as a function of the purification steps.

Dispersant	GGe	GTr	GTw
1 k rpm	14%	19%	26%
14 k rpm	13%	5%	7%

## Data Availability

The raw data and detailed methods can be obtained by contacting the corresponding authors.

## Supplementary Materials

The following supporting information can be downloaded at: https://www.mdpi.com/article/10.3390/s23167151/s1. References [7,35,39,40,41,42,43,44,45,46,87,88,89,90,91,92,93,94,95,96] are cited in the Supplementary Materials. Figure S1. Chemical structures of the dispersants used in the study: gelatin, triton X-100, and tween-20; Figure S2. (A) Digital photograph showing the AJP graphene sensor on a PET substrate with flexible screen-printed carbon electrodes. (B) Optical microscope image of the sensor surface; Figure S3. (A) Schematic representation of a printed graphene layer on a PET substrate topped with flexible screen-printed carbon electrodes. The dimensions of the flexible screen-printed carbon electrode and graphene film are shown. (B) An image of a graphene sample. (C) A homemade instrument used for measuring critical bending angles. (D, E) A custom-made device for measuring sample mechanical flexibility in both relaxed and bending states; Figure S4. An image of the screen-printed carbon electrochemical electrodes (BioDevice Technology, Ltd., Japan); Figure S5. AFM morphology images of a mica substrate surface with GGe at (A) low magnification and (B) high magnification, showing the gelation particle size accumulation on the mica substrate; Figure S6. TEM images of graphene sheets from (A–D) GGe, (E–H) GTr, and (I–L) GTw; Figure S7. Thermogram (TGA) and its derivative (DTG) for raw gelatin; Figure S8. Electrochemical analysis of GGe, GTr and GTw through (A–C) CV, (D–F) SWV, (G–I) EIS against [Fe(CN)_6_]^4−/3−^ electrolyte; Figure S9. Response curves of gas sensors printed with GGe, GTr, and GTw inks to (A) N2, (B) H2S, and (C) NH3 gases at 30% relative humidity and room temperature; Table S1. Properties of the dispersants provided by the chemical suppliers; Table S2. we have compiled a table summarizing the most recent publications on graphene inks printed using AJP; Table S3. Surface energy and HSPs of water, pristine graphene, GO and rGO.
