# Supplementary material for: Graphene Inks Printed by Aerosol Jet for Sensing Applications: The Role of Dispersant on the Inks’ Formulation and Performance
Source: Sensors (Basel). 2023 Aug 13;23(16):7151. doi: 10.3390/s23167151 (PMC10458541; doi:10.3390/s23167151)
Supplement: Supplementary file 1 [file sensors-23-07151-s001.zip › sensors-2554667-supplementary.pdf]

sensors

Supplementary Materials

Green Graphene Inks Printed by Aerosol-Jet for Sensing Applications: The Role of Dispersant on the Inks' Formulation and Performance

Ahmad Al Shboul [1,*], Mohsen Katabi [1], Daniella Skaf [2], Audithya Nyayachavadi [2], Thierry Lai [2], Tom Rautureau [2], Simon Rondeau-Gagné [2] and Ricardo Izquierdo [1,*]

[1] Department of Electrical Engineering, École de Technologie Supérieure, 1100, Rue Notre-Dame Ouest (Angle Peel), Montréal, QC H3C 1K3 Canada
[2] Department of Chemistry and Biochemistry, Advanced Materials Centre of Research, University of Windsor, Windsor, ON N9B 3P4, Canada
* Correspondence: ahmad.al-shboul@etsmtl.ca (A.A.S.); ricardo.izquierdo@etsmtl.ca (R.I.)

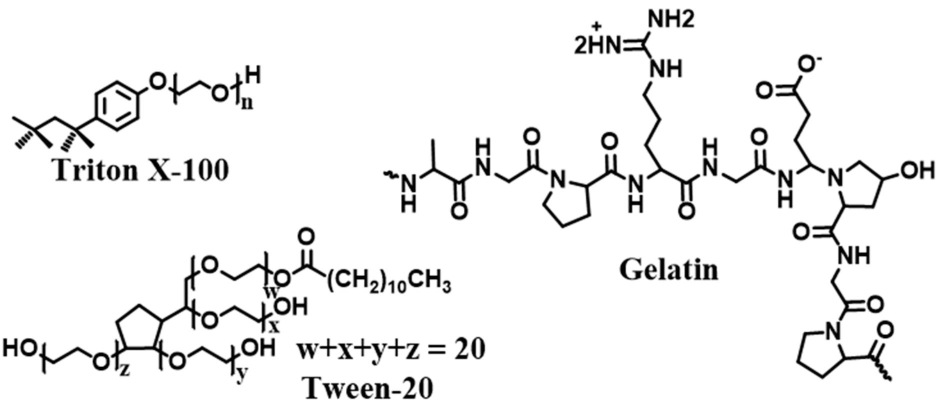

Figure S1. Chemical structures of the dispersants used in the study: gelatin, triton X-100, and tween-20.

Table S1. Properties of the dispersants provided by the chemical suppliers.

	Triton x-100	Tween-20	Gelatin
**Description**	Non-ionic	Non-ionic	Ionic/non-ionic
**Form**	Liquid	Liquid	Solid
**Density (g.cm^{-3})**	1.07	1.1	1.2
**M.Wt monomer**	625	1228	837
**Avg M.Wt**	80 k		50–60 k
**CMC (μM)**	200–900	40–60	12
**Solubility in water**	100 mg/mL	100 mg/mL	67 mg/mL @ 50 °C

Table S2. we have compiled a table summarizing the most recent publications on graphene inks printed using AJP. This table (Table S2) will be made available in the supporting information to complement and bolster our findings.

Ink	Solvent	Additives	Ink concentration	substrates	Printer nozzle (µm)	Application	Ref
**Graphene**	H_2O		~2.3 wt %	Kapton	150	Perfluoroalkyl substances (PFAS) Sensor	[1]
**Graphene**	H_2O			Kapton		Thin-film transistors	[2]
**Ag NPs-rGO/Ag**	H_2O/IPA/Terpineol	polyvinylpyrrolidone fluoropolymer	1 g/mL	Kapton	150	Temperature sensors	[3]
**Graphene**	Isobutyl acetate Diglyme, Glycerol Dihydrolevoglucosenone	Ethylcellulose Nitrocellulose	10 mg/mL		200	Pesticide parathion biochemical sensor	[4]
**Graphene**	H_2O Ethyl glycol	Polyvinylpyrrolidone	15%	Kapton	750	Hydrogen peroxide electrochemical sensor	[5]
**Graphene**	Ethanol Terpineol	Ethylcellulose	10 mg/mL	Kapton		SARS-CoV-2 Electrochemical sensor	[6]
**rGO and CNTs**	MeOH DMSO	PEDOT:PSS	0.5 mg/mL	Kapton,	1500	Stretchable supercapacitors	[7]
**Graphene**	Ethyl lactate and Terpineol	EC	30 mg/mL	Kapton	300	Thin-film transistors	[8]
**Commercial graphene ink**	H_2O	N/A		Paper	150	Lactate biosensor	[9]
**viral antigens-rGO**	H_2O	N/A	0.2 mg/mL	Glass	150	COVID-19 Antibodies sensor	[10]
**Graphene**	Dibutyl phthalate Ethyl lactate	Nitrocellulose	30 mg/mL	Kapton		Electrochemical histamine sensors	[11]
**Graphene**	Ethanol Terpineol	Ethylcellulose	10 mg/mL				[12]

Table S3. Surface energy and HSPs of water, pristine graphene, GO and rGO.

	Surface energy (mJ.m-2)	δ_D (MPa$^{1/2}$)	δ_P (MPa$^{1/2}$)	δ_H (MPa$^{1/2}$)	Ref
**Water**	72.7	15.6	16.0	42.3	[13–16]
**Graphene**	46.7	18	9.3	7.7	[17]
**GO**	54.8 - 62.1	17.1	10	15.7	[18,19]
**rGO**	23.4 – 46.7	16.7 - 17.49	9.49 - 10.7	14.1 - 14.41	[18–20]

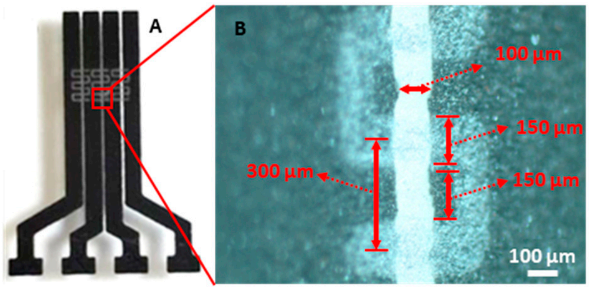

Figure S2. (A) Digital photograph showing the AJP graphene sensor on a PET substrate with flexible screen-printed carbon electrodes. (B) Optical microscope image of the sensor surface.

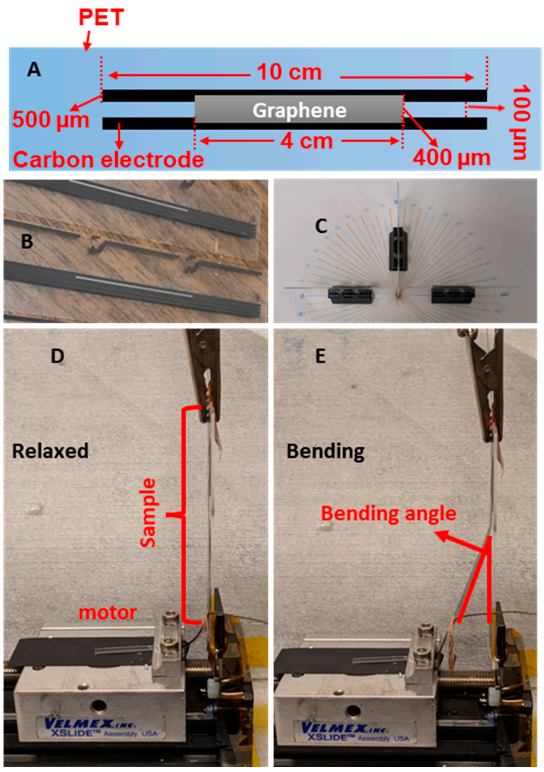

Figure S3. (A) Schematic representation of a printed graphene layer on a PET substrate topped with flexible screen-printed carbon electrodes. The dimensions of the flexible screen-printed carbon electrode and graphene film are shown. (B) An image of a graphene sample. (C) A homemade instrument used for measuring critical bending angles. (D, E) A custom-made device for measuring sample mechanical flexibility in both relaxed and bending states.

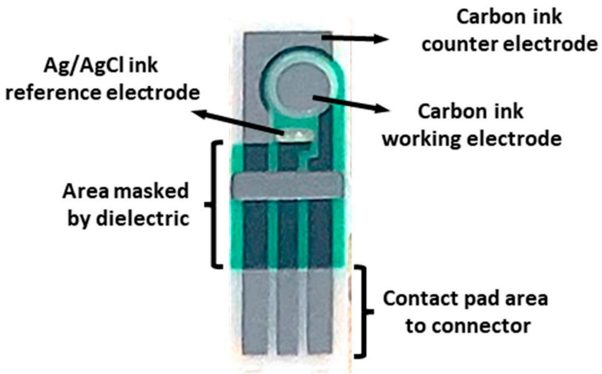

Figure S4. An image of the screen-printed carbon electrochemical electrodes (BioDevice Technology, Ltd., Japan).

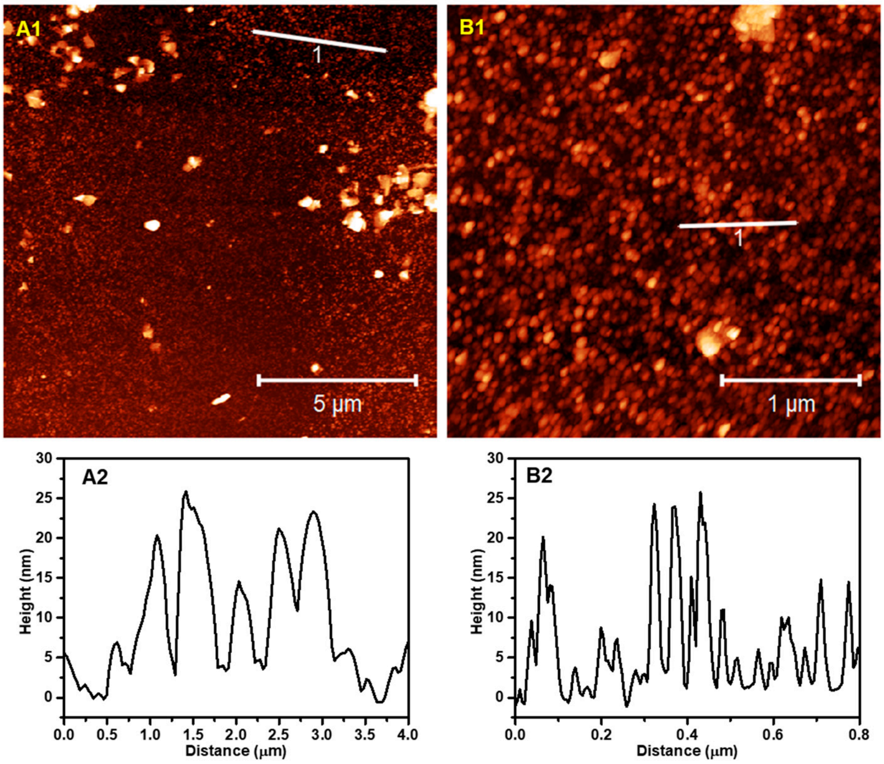

Figure S5. AFM morphology images of a mica substrate surface with GGe at (A) low magnification and (B) high magnification, showing the gelation particle size accumulation on the mica substrate.

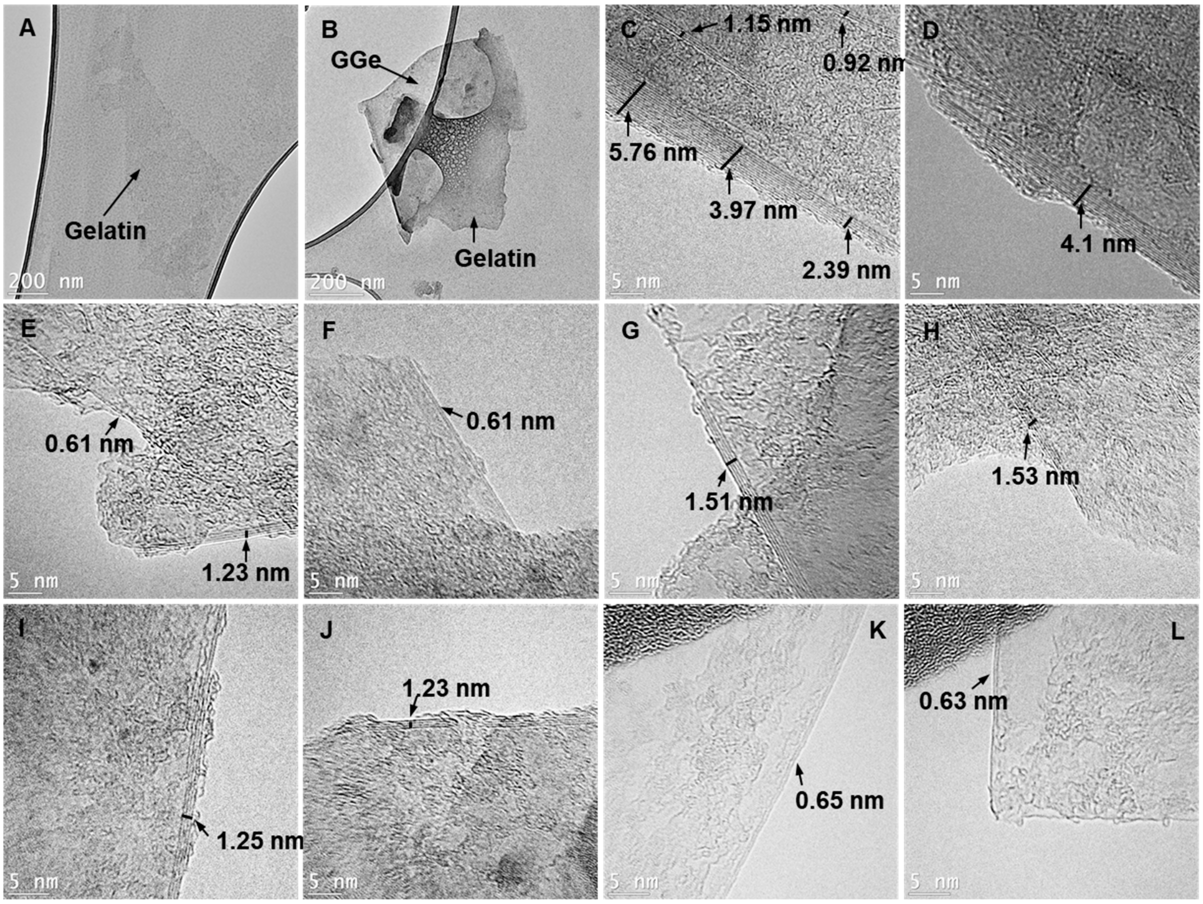

Figure S6. TEM images of graphene sheets from (A-D) GGe, (E-H) GTr, and (I-L) GTw.

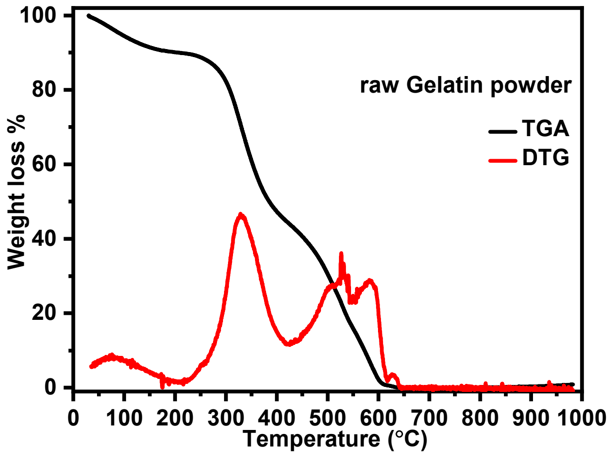

Figure S7. Thermogram (TGA) and its derivative (DTG) for raw gelatin.

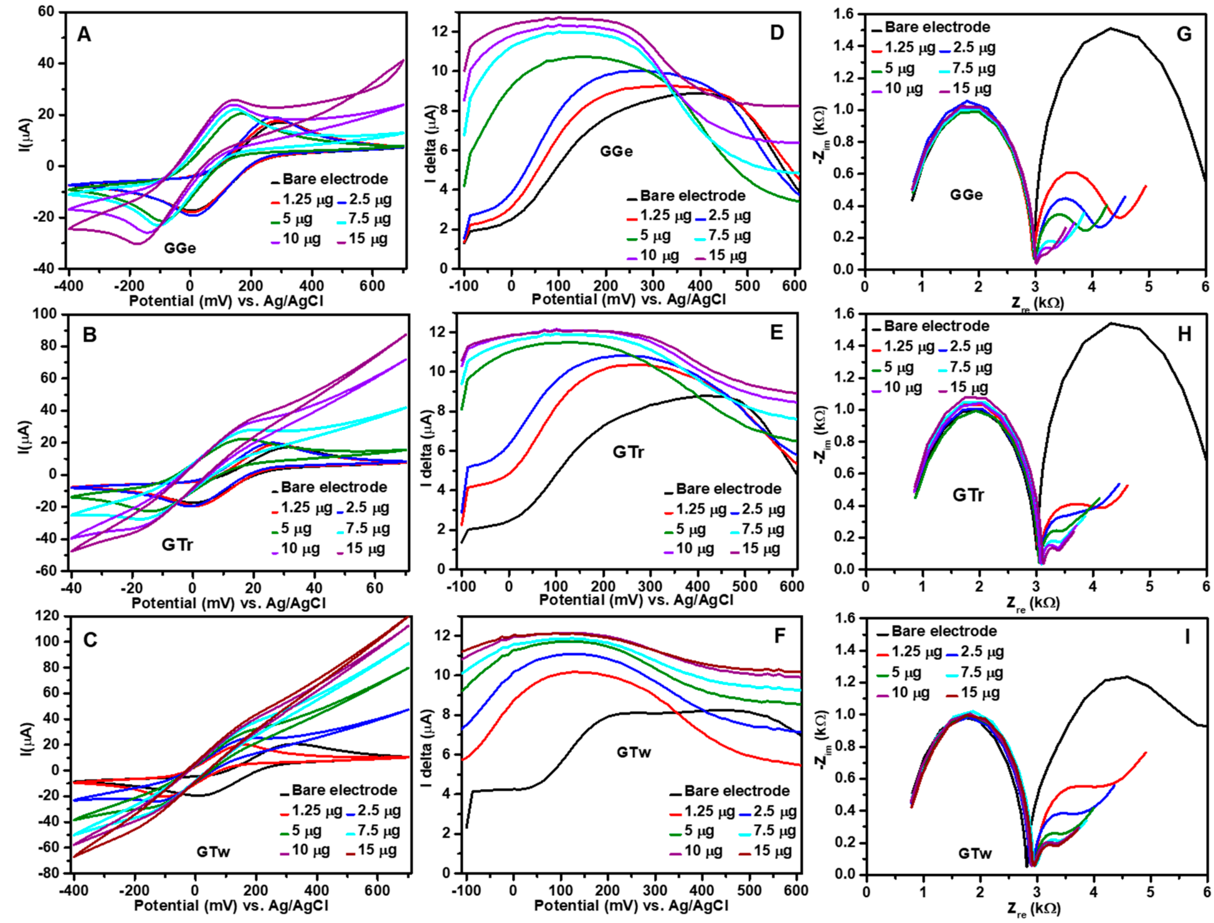

Figure S8. Electrochemical analysis of GGe, GTr and GTw through (A,B,C) CV, (D,E,F) SWV, (G,H,I) EIS against $[Fe(CN)_6]^{4-/3-}$ electrolyte.

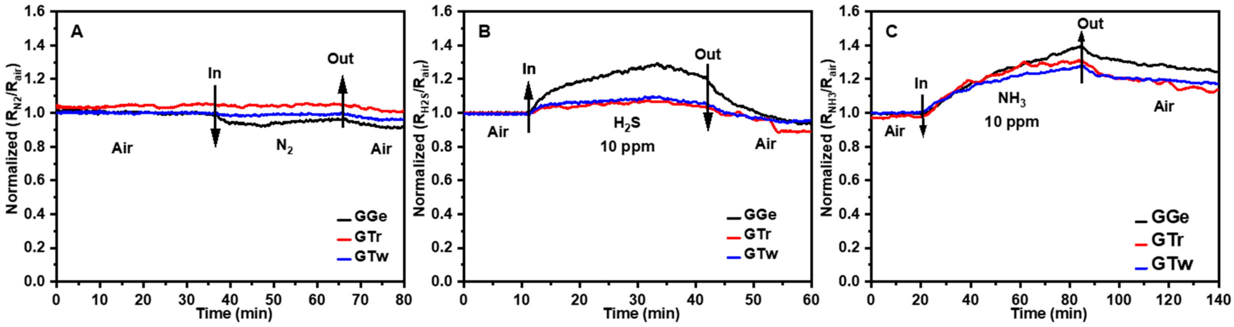

Figure S9. Response curves of gas sensors printed with GGe, GTr, and GTw inks to (A) N_2, (B) H_2S, and (C) NH_3 gases at 30% relative humidity and room temperature.

References

1. McDonnell, C.; Albarghouthi, F.M.; Selhorst, R.; Kelley-Loughnane, N.; Franklin, A.D.; Rao, R. Aerosol Jet Printed Surface-Enhanced Raman Substrates: Application for High-Sensitivity Detection of Perfluoroalkyl Substances. *ACS Omega* **2023**, *8*, 1597–1605, doi:10.1021/acsomega.2c07134.
2. Lu, S.; Smith, B.N.; Meikle, H.; Therien, M.J.; Franklin, A.D. All-Carbon Thin-Film Transistors Using Water-Only Printing. *Nano Lett.* **2023**, *23*, 2100–2106, doi:10.1021/acs.nanolett.2c04196.
3. Niu, Y.; Han, Y.; Cheng, H.; Xiong, Z.; Luo, B.; Ma, T.; Li, L.; Liu, S.; Chen, X.; Yi, C. Synthesized Silver Nanoparticles Decorated Reduced Graphene Oxide/Silver Ink for Aerosol Jet Printed Conformal Temperature Sensor with a Wide Sensing Range and Excellent Stability. *J. Mater. Res. Technol.* **2023**, *25*, 873–886, doi:10.1016/j.jmrt.2023.05.246.
4. Gamba, L.; Johnson, Z.T.; Atterberg, J.; Diaz-Arauzo, S.; Downing, J.R.; Claussen, J.C.; Hersam, M.C.; Secor, E.B. Systematic Design of a Graphene Ink Formulation for Aerosol Jet Printing. *ACS Appl. Mater. Interfaces* **2023**, *15*, 3325–3335, doi:10.1021/acsami.2c18838.
5. Fapanni, T.; Sardini, E.; Serpelloni, M.; Tonello, S. Nano-Functionalized Electrochemical Sensors by Aerosol Jet Printing. *IEEE Sens. J.* **2022**, *22*, 21498–21507, doi:10.1109/JSEN.2022.3213349.
6. Pola, C.C.; Rangnekar, S. V.; Sheets, R.; Szydłowska, B.M.; Downing, J.R.; Parate, K.W.; Wallace, S.G.; Tsai, D.; Hersam, M.C.; Gomes, C.L.; et al. Aerosol-Jet-Printed Graphene Electrochemical Immunosensors for Rapid and Label-Free Detection of SARS-CoV-2 in Saliva. *2D Mater.* **2022**, *9*, 035016, doi:10.1088/2053-1583/ac7339.
7. Zhou, Y.; Parker, C.B.; Joshi, P.; Naskar, A.K.; Glass, J.T.; Cao, C. 4D Printing of Stretchable Supercapacitors via Hybrid Composite Materials. *Adv. Mater. Technol.* **2021**, *6*, 2001055, doi:10.1002/admt.202001055.
8. Hyun, W.J.; Chaney, L.E.; Downing, J.R.; De Moraes, A.C.M.; Hersam, M.C. Printable Hexagonal Boron Nitride Ionogels. *Faraday Discuss.* **2021**, *227*, 92–104, doi:10.1039/c9fd00113a.
9. Williams, N.X.; Bullard, G.; Brooke, N.; Therien, M.J.; Franklin, A.D. Printable and Recyclable Carbon Electronics Using Crystalline Nanocellulose Dielectrics. *Nat. Electron.* **2021**, *4*, 261–268, doi:10.1038/s41928-021-00574-0.
10. Ali, M.A.; Hu, C.; Jahan, S.; Yuan, B.; Saleh, M.S.; Ju, E.; Gao, S.J.; Panat, R. Sensing of COVID-19 Antibodies in Seconds via Aerosol Jet Nanoprinted Reduced-Graphene-Oxide-Coated 3D Electrodes. *Adv. Mater.* **2021**, *33*, 1–15, doi:10.1002/adma.202006647.
11. Parate, K.; Pola, C.C.; Rangnekar, S. V.; Mendivelso-Perez, D.L.; Smith, E.A.; Hersam, M.C.; Gomes, C.L.; Claussen, J.C. Aerosol-Jet-Printed Graphene Electrochemical Histamine Sensors for Food Safety Monitoring. *2D Mater.* **2020**, *7*, doi:10.1088/2053-1583/ab8919.
12. Tafoya, R.R.; Cook, A.W.; Kaehr, B.; Downing, J.R.; Hersam, M.C.; Secor, E.B. Real-Time Optical Process Monitoring for Structure and Property Control of Aerosol Jet Printed Functional Materials. *Adv. Mater. Technol.* **2020**, *5*, 1–6, doi:10.1002/admt.202000781.
13. Barton, A.F.M. *CRC Handbook of Solubility Parameters*; 1983; ISBN 9780849332951.
14. Shin, K.-Y.; Hong, J.-Y.; Jang, J. Micropatterning of Graphene Sheets by Inkjet Printing and Its Wideband Dipole-Antenna Application. *Adv. Mater.* **2011**, *23*, 2113–2118, doi:10.1002/adma.201100345.
15. Carl L. Yaws *Thermophysical Properties of Chemicals and Hydrocarbons 1st Edition*; 2008; ISBN 9780815519904.
16. The Chemical Rubber Company *CRC Handbook of Chemistry and Physics, 50th Edition*; Weast, R.C., Ed.; 50th ed.; Cleveland, 1969;
17. Al Shboul, A.; Trudeau, C.; Cloutier, S.; Siaj, M.; Claverie, J.P. Graphene Dispersions in Alkanes: Toward Fast Drying Conducting Inks. *Nanoscale* **2017**, *9*, 9893–9901, doi:10.1039/C7NR01919J.
18. Konios, D.; Stylianakis, M.M.; Stratakis, E.; Kymakis, E. Dispersion Behaviour of Graphene Oxide and Reduced Graphene Oxide. *J. Colloid Interface Sci.* **2014**, *430*, 108–112, doi:10.1016/j.jcis.2014.05.033.
19. Wan, D.; Yang, C.; Lin, T.; Tang, Y.; Zhou, M.; Zhong, Y.; Huang, F.; Lin, J. Low-Temperature Aluminum Reduction of Graphene Oxide, Electrical Properties, Surface Wettability, and Energy Storage Applications. *ACS Nano* **2012**, *6*, 9068–9078, doi:10.1021/nn303228r.
20. Ayán-Varela, M.; Paredes, J.I.; Villar-Rodil, S.; Rozada, R.; Martínez-Alonso, A.; Tascón, J.M.D. A Quantitative Analysis of the Dispersion Behavior of Reduced Graphene Oxide in Solvents. *Carbon N. Y.* **2014**, *75*, 390–400, doi:10.1016/j.carbon.2014.04.018.
